# Sex as a risk factor for occurrence and severity of infectious and parasitic diseases in dogs: Protocol for a systematic review

**DOI:** 10.1371/journal.pone.0275578

**Published:** 2022-10-25

**Authors:** Charles Byaruhanga, Darryn Knobel

**Affiliations:** 1 Faculty of Veterinary Science, Department of Veterinary Tropical Diseases, University of Pretoria, Pretoria, South Africa; 2 Center for Conservation Medicine and Ecosystem Health, Ross University School of Veterinary Medicine, Basseterre, Saint Kitts, West Indies; Beni Suef University Faculty of Veterinary Medicine, EGYPT

## Abstract

Biological sex is an important risk factor for the occurrence and severity of infectious and parasitic diseases. Although various studies and reviews have described sex differences in infectious diseases of humans, wildlife and laboratory animals, there has been little focus on biological sex as a risk factor for infectious and parasitic diseases of domestic animals. We aim to identify and synthesise evidence in dogs for the hypothesis that biological sex and gonadectomy status are determinants of occurrence and severity of disease across taxa of pathogens. This systematic review follows the Preferred Reporting Items for Systematic reviews and Meta-analyses (PRISMA) guidelines. We will search Web of Science, Scopus and PubMed for peer-reviewed studies published in English from database inception through 2021. All study designs for infectious and parasitic diseases of dogs will be included. This review will include the outcomes prevalence or incidence of infection or disease; and severity of disease as measured by case-fatality, time to death or recovery, hospitalisation time, pathogen burden (e.g. viral load or parasitaemia) or relevant clinicopathological parameters. Two reviewers will jointly assess the first 500 records from all three databases. Subsequently, one reviewer will screen the remaining records, and then the second reviewer will verify all records excluded by the first reviewer. Full-texts of all included records will be retrieved and assessed for eligibility by the first review author, and then the second author will review those records excluded by the first author. The risk of bias in individual studies will be assessed using the Risk of Bias Assessment tool for Nonrandomized Studies. We will synthesise the information from the studies and present this as a narrative in the text. The findings will be presented by outcome type and also grouped by pathogen type. Evidence on sex-specific effects will expand our understanding of infectious disease pathogenesis and underlying mechanisms, and this may be of importance in implementation of disease control interventions.

## 1. Introduction

Infectious and parasitic diseases are widespread across animal species, but the patterns of infection vary between populations and between individuals in a population [1, 2]. Differences in infection patterns and outcomes can be attributed to exposure factors, such as differential contact with pathogens through ecological and behavioral differences, or due to differences in disease susceptibility that are mediated by factors such as hormones, age or stress [1]. Sex differences have been documented, but mostly in humans [3–5], non-domestic animals [2] and murine models [6, 7], and the general observation is that males are more susceptible to a diversity of infectious and parasitic diseases than females. The effect of sex manifests as higher prevalence and severity of infectious diseases in males; however, higher rates of autoimmune diseases manifest in females [8, 9].

Sex-related differences in infectious and parasitic disease among vertebrate and invertebrate animals have been attributed to differences in immune responses, with females considered to be more ‘immunocompetent’ [2, 10, 11] and males considered to be the ‘sicker sex’ [12]. The superior immune responsiveness of females manifests as higher levels of antibodies (IgM, IgG, IgA) and stronger cell-mediated and humoral immunity [13, 14]. The differences in immune responses could be due to differences in the expression of genes encoded on the X and Y chromosomes [15] or differences in sex hormones that regulate the immune system [16]. Testosterone, for example, has immunosuppressive effects in males and estrogen has positive effects in females [11, 16, 17], while higher white blood cell counts in females are associated with longevity [11]. It is argued that females invest more resources into immune defenses than males and this compensates for the physiological stress that comes with reproduction, thus ensuring increased longevity and reproductive success [10, 11, 18]. Although various studies report superior female immunocompetence, other reports indicate male-biased immunocompetence, or no difference between males and females [19–22]. Even then, the superior immunocompetence in females is not consistent across all animal species, but differs by taxonomic classification. Larger effect sizes were reported in insects than higher level animals [2], supporting the notion that testosterone is not the only determinant of sex differences in immunity between sexes. Age-related effects are also predicted to occur if sex differences in immunity are due to differential resource allocation as a trade-off with reproduction [2], waning immune function [23, 24], or to cumulative pathogen exposure over time [25]. Kelly et al. [2] found that female-biased immunity was more pronounced in adult than immature animals.

These sex differences in immunity are likely to cause variation in disease occurrence and severity [4] between individuals in animal populations with implications for disease control, for example treatment or vaccination based on sex predisposition, and selection for superior performance and disease tolerance/resistance. However, despite the growing recognition of sex as a determinant for disease susceptibility in humans [5, 26, 27], and the calls to include sex as a biological variable in treatment and design of studies [28, 29], there seems to be little attention regarding the role of sex in domestic animal infections. In many instances, there is no deliberate consideration of sex as a biological variable in investigating outcomes in pathogenesis and clinical studies in animals [30]. Some studies and reviews on animals have explored sex differences in immunity, but mostly among non-domestic animal species [2]. Despite the wealth of veterinary studies, there has been little systematic focus on sex differences in occurrence or severity of infectious and parasitic diseases among domestic animal species.

In this systematic review, we will identify and summarise available evidence of sex as a risk factor for the occurrence and severity of infectious and parasitic diseases in dogs. We have elected to focus on dogs because of the large numbers of studies on the occurrence of infectious and parasitic diseases in this species, and the fact that surgical sterilisation (gonadectomy) is commonly undertaken for reproductive management of this species. This provides opportunity to examine evidence for the effect of sex as well as gonadectomy on occurrence and severity of disease. Evidence on sex-specific effects will expand our understanding of infectious disease pathogenesis and underlying mechanisms, and demonstrate the importance of considering sex in the design and interpretation of infectious disease studies. Findings may also be of importance in implementation of interventions to prevent or manage disease.

### 1.1 Review questions

- Is sex (biological sex and gonadectomy status) a determinant of occurrence (incidence or prevalence) and severity of infectious and parasitic diseases in dogs?
- Do sex differences in occurrence or severity of infectious and parasitic diseases differ across taxa of pathogens?
- Is there evidence that sex differences in these diseases are due to differing immunological responses between the sexes?

### 1.2 Aim

The aim of the present systematic review is to identify, summarise and synthesise the findings of published studies on sex-related susceptibility to infection and disease between female and male dogs and considering their status as sterilised, unsterilised or unknown.

### 1.3 Objectives

- Investigate sex-related differences in terms of occurrence, severity and immune responses across a range of infectious and parasitic diseases of dogs.
- Discuss underlying mechanisms of sex-differential susceptibility.
- Identify gaps that can contribute to planning, design and implementation of future research on sexual dimorphism in immune function in companion animals

## 2. Materials and methods

This systematic review protocol is prepared following the Preferred Reporting Items for Systematic reviews and Meta-Analyses Protocols (PRISMA-P) (www.prisma-statement.org) 2015 statement [31], PRISMA-P Explanation and Elaboration [32] and updated PRIMSA 2020 explanation and elaboration [33]. The checklist for the protocol is presented in S1 File. The review is supported by funding from the National Institute of Allergy and Infectious Diseases of the National Institutes of Health (award number R21AI151356) and the University of Pretoria (award number UP/13399650). The University of Pretoria and Ross University School of Veterinary Medicine are providing administrative support for the systematic review. Moreover, the University of Pretoria library will provide a platform for literature search, access to the various databases and the data management software (EndNote). These institutions have no role in the design, collection, analysis or interpretation of the data, as well as writing and submission of the manuscript for publication. The study will be registered on PROSPERO–International Prospective Register of Systematic Reviews soon after peer review. The protocol began on September 1, 2021 and the systematic search for literature will be conducted from January through August 2022. The expected completion date of the systematic review is December 31, 2022.

### 2.1 Ethical approval and dissemination

This study will not involve primary data collection or animal research, and therefore no formal ethics approval is required. The findings will be published in a peer-reviewed journal and presented at conferences or through other popular media.

### 2.2 Eligibility criteria (Exclusion and inclusion criteria)

The search process will broadly include all studies that report occurrence or severity of infectious or parasitic diseases by sex in dogs, and where possible consider the animals’ status as sterilised, unsterilised or unknown. We will consider epidemiological, clinical or experimental studies of infections in dogs. We will include data for all age categories of dogs if reported separately. Studies that describe susceptibility related to anatomical differences between the sexes will be excluded from analysis. The assessment criteria will be based upon four main components (i) language: only studies written in English will be searched; (ii) study type: original peer-reviewed journal article or study (excluding case reports) and postgraduate dissertations/theses published from database inception through December 2021; (iii) study topic: if there is information by sex for infectious and parasitic diseases of dogs and (iv) study design: both observational and experimental studies will be included. No limit will be placed on geographical region. We will not search non-peer reviewed documents (e.g. commentaries, newsletters) and other grey literature. Conference proceedings and books/book chapters will also be excluded. Studies on ectoparasites as well as autoimmune diseases will be excluded. Review articles and meta-analyses will only be included if they contain relevant citations for further reading.

### 2.3 Search methods

#### 2.3.1 Information sources

The systematic search for literature will be conducted using the electronic book Greene’s Infectious Diseases of the Dog and Cat [34] (https://www.elsevier.com/books/infectious-diseases-of-the-dog-and-cat/sykes) to scope for infectious diseases of dogs, followed by searching three electronic databases: PubMed (1940 through 2021), Scopus (Elsevier, 1960 through 2021) and ISI Web of Science (Clarivate Analytics, 1945 through 2021) for all references that report occurrence or severity of infectious or parasitic diseases of dogs by sex. These databases will be searched for a wide range of diseases of dogs caused by helminth, viral, bacterial, rickettsial, chlamydial, mycoplasmal, fungal and protozoal pathogens. On the Web of Science, we will select the option ‘all databases’ so as to retrieve literature from all databases on this platform, including CAB Abstracts and Global Health, MEDLINE and Zoological Records. In case a full-text of an eligible study is not available on the University of Pretoria platform, we will contact other libraries to share the document with us.

#### 2.3.2 Search strategy and search terms

The search strategy is drafted and refined by the two authors of this protocol (CB, DK), who have experience in systematic and/or scoping reviews, in consultation with a University of Pretoria Veterinary Sciences information specialist with expertise in systematic review searching. The search terms are categorised under three main search term topics: 1) animal species, 2) sex-related, and 3) diseases. We have developed a list of search terms considered as sex-related: “sex”, “male”, “gender”, “castrat*”, “spay”, “ovariohysterectom*”, “gonadectom*”, “orchidectom*”, “steril*”, “neuter”. Some of the terms have been truncated to broaden the search output, for example “steril*” will be used to pick up “sterile” as well as “sterilisation”, “castrat*” to pick up “castrate” and “castration”, and “male” to pick up the term “female” as well. Although gender refers to social, behavioral, cultural and psychological aspects of males and females (humans in particular), some studies incorrectly use sex and gender interchangeably. We therefore include both sex and gender in our search strategy, although our interest is in the former. The disease categories included are viral, bacterial, rickettsial, chlamydial, mycoplasmal, fungal, protozoal and helminths, and these have been truncated to broaden the search.

The final search strategy for the studies in the three electronic databases is found in S2 File. The Boolean operators “AND” and “OR” will be used between topic groups and search terms respectively.

## 2.4 Study selection

Study selection will proceed in stages. In Stage 1, references (title and abstract) that are selected based on the search terms will be imported into EndNote^TM^ 20 (Philadelphia, PA, United States of America) by CB. Duplications will be removed using a built-in function and further checked manually during subsequent screening stages. We will avoid double reporting on studies that may be published in more than one form (e.g. journal article and thesis) by screening author names associated with multiple publications in the data extraction form. If reports on the same study supplement each other in terms of content, all will be considered.

In Stage 2, both reviewers (CB and DK) will jointly assess the first 500 records from all electronic database using titles and abstracts. Subsequently, CB will screen the remaining records (titles and abstracts), and then DK will review all records excluded by CB. Any discrepancies will be resolved by discussion and mutual agreement of the two authors. When eligibility cannot be established by reading the title or abstract, the full-text will be retrieved and screened in the third stage. In Stage 3, CB will retrieve the full text of records included from Stage 2 and assess these for eligibility, and then DK will review the full text of those records excluded by CB. Any discrepancies will be resolved as above. In Stage 4, both CB and DK will independently review the full text of all records included from Stage 3 and finalise the list of included records. Subsequently, CB will extract data from the included records, and then DK will check the collected data. Any discrepancies in the included records and extracted data at Stage 4 will be resolved through discussions to reach a consensus.

### 2.5 Data extraction and management

The two reviewers (CB, DK) will jointly develop a standardised data extraction form in Microsoft Excel® (Microsoft Corporation, Redmond, WA, United States), which will be calibrated for comprehensive and accurate capture and management of relevant information from each article or study retrieved from the three electronic databases. The form will include study characteristics and information, including author(s), publication year, journal, title, country, objectives, study/experimental population context, pathogen species, disease, intervention strategies, measured parameters and key data and conclusions on sex-related differences in occurrence or severity of diseases of dogs.

If available, we will extract disease parameters by sex, including severity, prevalence, incidence and duration of symptoms and mortality, as well as underlying mechanisms such as immune response, hormonal expression and genetic factors. Whenever available, we will extract study design, study size and subject characteristics (e.g. age, sterilisation status). Unclear or incomplete information in an article/study will be dealt with by searching other articles cited or any other sources of information. In circumstances where authors do not specify the sterilisation status, we will take this as ‘unknown status.’

### 2.6 Outcomes and prioritisation

The main outcomes for this systematic review are prevalence or incidence of infection or disease; and severity of disease as measured by case-fatality, time to death or recovery, hospitalisation time, pathogen burden (e.g. viral load or parasitaemia) or relevant clinicopathological parameters. In studies that report disease occurrence or severity by age, we will subdivide and report the sex-related differences as such.

### 2.7 Risk of bias in individual studies

The risk of bias for individual studies will be assessed using the Risk of Bias Assessment Tool for Nonrandomized Studies (RoBANS; [35]). The six domains to be evaluated are participant selection, confounding variables, measurement of exposure, blinding of outcome assessment, incomplete outcome data and selective outcome reporting [35]. The two authors (CB, DK) will independently evaluate each included study and record judgements concerning risk of bias for each domain as ‘low risk ‘, ‘moderate risk’ or ‘high risk’ [35]. If there is inadequate data reported in the study, we will classify the risk of bias as ‘unclear’. We will report on all studies regardless of risk of bias provided that there is sufficient data on occurrence or severity of disease by sex of dogs. Disagreements in scoring between the two authors will be settled through discussions to reach a consensus.

### 2.8 Data synthesis

We will synthesise the information from the different studies and present this as a narrative in the text, as described by the PRISMA-P Group [33], highlighting the extent and nature of evidence available regarding disease-related sex dimorphism in dogs.

We will present our findings by outcome, being either occurrence or severity, and then group these by pathogen type. The systematic narrative synthesis will summarise the relative contribution of pathogen types (viral, bacterial, rickettsial, chlamydial, mycoplasmal, protozoal, fungal, helminth) and underlying mechanisms (e.g. hormonal, genetic) across the included studies. This will be followed by analysis of the main and additional sex-differential outcome measures grouped by infection type, and the underlying mechanisms. By assessing the studies within key dimensions, we can systematically assess the commonalities in terms of how much literature is available, and to an extent explain the discrepancies within and across dimensions.

Lastly, we will provide an overall picture of the evidence available on sex-differential infectious disease susceptibility, and a description of the implications for animal health and welfare management, future research and policy. The systematic review will end with an outline of the important gaps in the evidence available and recommendations for direction to basic and applied research.

### 3. Conclusion

Sex differences in infectious diseases can vary significantly across animal species and pathogen types and various mechanisms may be responsible for the differences. This study will examine the evidence for sex differences in occurrence and severity of infectious diseases among dogs and the role that hormonal and genetic factors may play in immune responses. The conclusion will stem from synthesis of sex-related information regarding dogs from various studies. Where adequate data are available, we will perform meta-analyses in future studies to confirm the effect of sex for selected diseases or pathogen types. Further assessment of the affected age groups of both sexes and sex-specific life history (e.g. sterilisation status) can also contribute to understanding the regulation of immune response by sex hormones.

A limitation of this study is that the literature search will be restricted to articles published in the English language, and this may miss out relevant articles published in other languages.

## Supporting information

S1 File PRISMA-P 2021 checklist. (DOCX)

S2 File Search terms. (DOCX)
