# Supplementary material for: Sex as a risk factor for occurrence and severity of infectious and parasitic diseases in dogs: Protocol for a systematic review
Source: PLoS One. 2022 Oct 25;17(10):e0275578. doi: 10.1371/journal.pone.0275578 (PMC9595549; doi:10.1371/journal.pone.0275578)
Supplement: S2 File — (DOCX) [file pone.0275578.s002.docx]

**Appendix 2**. List of terms to be used in search for studies in a systematic literature review on the occurrence and severity of infectious and parasitic diseases by sex in dogs. The Boolean ‘AND” will be included between the search term groups

| **Groups** | **Topic** | **List of terms** |
| --- | --- | --- |
| Group 1 | Animal species | (dog OR canine) |
| Group 2 | Sex-related | (sex OR male OR gender OR castrat* OR spay OR ovariohysterectom* OR gonadectom* OR orchidectom* OR steril* OR neuter) |
| Group 3 | Diseases | (infectio* OR virus OR viral OR bacteri* OR rickettsi* OR chlamydia* OR mycoplasma* OR fung* OR protozoa* OR helminth* OR worm)) |
